# Experiences of Negotiations for Improving Research Environment and Burnout among Young Physician Researchers in Japan

**DOI:** 10.3390/ijerph17145221

**Published:** 2020-07-20

**Authors:** Masato Toyoshima, Shinichi Takenoshita, Hitoshi Hasegawa, Takuma Kimura, Kyoko Nomura

**Affiliations:** 1 Akita Prefectural Daisen Public Health Center, 13-62 Omagari Kamisakae-cho, Daisen, Akita 014-0062, Japan; Toyoshima-Masato@pref.akita.lg.jp; 2 Department of Public Health, Akita University Graduate School of Medicine, 1-1-1 Hondo, Akita 010-8543, Japan; shin.takeno@gmail.com; 3 Department of Medical Education, Akita University Graduate School of Medicine, 1-1-1 Hondo, Akita 010-8543, Japan; hasegawa@doc.med.akita-u.ac.jp; 4 Department of General Medicine, Saitama Medical University, 38 Morohongo, Moroyama-machi, Iruma-gun, Saitama 350-0495, Japan; takuma7kimura@gmail.com

**Keywords:** burnout, cross-sectional study, mental well-being, negotiation experience, physician researcher, research environment

## Abstract

Physician scientists in Japan are often too busy to be sufficiently involved in research work. This cross-sectional study aimed to investigate their experiences negotiating with their superiors to improve their research environment and determine its relationship with psychological burnout. Among 1790 physician awardees of Grants-in-Aid for Young Scientists in 2014–2015, 490 responded (response rate 27.4%) and 408 were eligible for analysis. Outcome measures included two negotiation experiences: for reduction of clinical duty hours/promotion opportunities and for increased space or equipment/increased research budget. The main explanatory variables were personal, patient-related, and work-related burnout measured by the Copenhagen Burnout Inventory. The percentages of the above-mentioned two types of negotiations were 20–24% in women and 17–20% in men. Multivariable stepwise logistic regression analyses demonstrated that (1) the negotiation for reduction of clinical duty hours/promotion opportunities was significantly associated with physician scientists who had a short amount of weekly research time and high patient-related burnout score, and (2) the negotiation for increased space or equipment/increased research budget was significantly associated with older age, single status, and high personal and patient-related burnout scores. High burnout is related to negotiation experiences among physician researchers in Japan.

## 1. Introduction

Research conducted by physicians is indispensable for advancements in the quality of medicine because an original idea is usually embedded in daily practice. However, the share of papers on clinical medicine and basic life sciences from Japan has decreased since around the year 2000 [1]. Japan now has only a 6.0% and 5.5% share (2015–2017) in the number of papers on clinical medicine and basic life sciences, respectively [1].

One reason for the decrease in the number of Japanese papers may be because the Grant-in-Aid for Scientific Research (KAKENHI) is no longer sufficient. KAKENHI has declined after peaking in 2011 (263,300 million yen in 2011 vs. 228,600 million yen in 2018). Moreover, the small share of scientific output from Japan may be explained by the limited protected time for research due to physician shortages in clinical practice. In Japan, the number of medical doctors per unit population has been the lowest among Organisation for Economic Co-operation and Development (OECD) countries for over three decades; thus, Japanese licensed medical doctors have voluntarily accepted overwork [2]. Burnout is common worldwide in practice settings where physicians are forced to work long hours [3,4,5,6]. Previous studies have suggested that about half of physicians are affected by symptoms of burnout [7,8]. A cross-sectional study in Brazil suggests that higher burnout is associated with being pessimistic, working in an ICU or emergency department, perceiving a lack of hospital recognition, and excess work [5]. Another cross-sectional study in New Zealand suggests that high burnout is associated with poor health status, working more than 14 consecutive hours, and being a woman [3]. Cross-sectional studies in the US suggest that higher burnout is associated with younger age, having children, lower quality of life (QOL), greater educational debts, long working hours, poor working environment, and less time spent on the most meaningful activities [6,7,9,10]. Interestingly, physician burnout may sometimes affect academic outputs. A cross-sectional study in Sweden suggests that burnout has a negative impact on the number of published articles among female physicians [11]. In an environment of increasing workload, there may be additional pressure for physician researchers to conduct research in a short time. Such a limited protective time may decrease the motivation of physicians for engaging in research. In Japan, when the new postgraduate medical education (PGME) system along with the National Resident Matching Program was introduced in 2004 [12,13], half of the residents are more likely to shift their residency site from university to non-university hospitals [14]. This may be related to the fact that young physicians are not enthusiastic to obtain a PhD (28% for women and 40% for men), which is awarded by a university, but desirous of acquiring the title of ‘specialist,’ which is awarded by the Society of Various Natural Science (95% for women and 93% for men) [15].

Negotiation behaviors among physician researchers are a useful method for improving their research environment as well as mental well-being [16], but it has been little studied. Resources such as laboratory space, funding, personnel, and protected research time are vital components to academic success. Many medical faculty members have little awareness of the importance of negotiation and are not trained in how to negotiate with a responsible person in their workplace [17]. Creating favorable environments for young physician scientists is important for their academic career along with their duties as a practicing physician. Therefore, it may contribute to their psychological well-being. The purpose of this study is to investigate whether physician researchers’ negotiations with responsible persons to improve their research environment relate to their mental well-being. As negotiating their working conditions with their senior faculty or superiors who are key persons at their worksite is absolutely necessary, the result of the present study may be helpful to find a way to balance clinical and research work and improve the quality of healthcare.

## 2. Materials and Methods

### 2.1. Participants

The Grant-Aid for Scientific Research Database was used to identify 3143 awardees of KAKENHI for Young Scientists in 2014 and 2015. The Ministry of Health, Labor, and Welfare doctor’s license database was then used to identify medical doctors among the awardees. The eligible population consisted of 1739 medical doctors aged between 29 and 41 years old, excluding scientists not registered in the doctors’ license database and those who withdrew from the grant. We then conducted an internet search to obtain mailing addresses for 1739 medical doctors to recruit and send a questionnaire. Of these, 490 (response rate, 28.1%) responded with written informed consent. We excluded respondents who did not answer the question of whether they held a medical doctor’s license as well as those who were not engaged in a clinical practice. These exclusions left 408 (23.4%) physician scientists as the final sample.

This study was approved by the Teikyo University ethics committee (TU-COI 13-208).

### 2.2. Questionnaire

The variables measured in this study included age, gender, grant-in-aid for young scientists, position/post at work, weekly working time attributes, self-rated sufficiency of research environment, negotiation experience, and burnout. There are two types of grants-in-aid for young scientists: Type A (5–30 million yen research grant) and Type B (less than 5 million yen research grant). Position was classified into six categories: associate professor, lecturer, assistant professor, administrator/director, registered doctor, and resident/research associate/other. Self-rated sufficiency of research environment was answered using a five-point Likert scale ranging from 5 points for “sufficient” to 1 point for “insufficient.” To measure negotiation experience, we asked whether, in the past two years, the participant had asked a superior at their institution for the following four improvements (yes/no for each experience): reduce amount of clinical duty hours, have more spacious room or equipment, increase research budget, and have opportunities for promotion. Degree of burnout was assessed using a translated Japanese version of the Copenhagen Burnout Inventory (CBI) [18,19]. The CBI, which has recently been used to measure burnout among medical physicians [20,21], is a nineteen-item survey for assessing three burnout subscales: personal (6 items), work-related (7 items), and client-related burnout (6 items). Cronbach’s alpha was estimated for each subscale. A total of 19 items were answered using a five-point Likert scale ranging from 100 points for “always/to a very high degree” to 0 points for “never/to a very low degree”. The three burnout subscale scores were calculated by averaging all relevant items, with higher overall scores indicating a higher degree of burnout.

### 2.3. Statistical Analysis

We first grouped the four negotiations into two categories according to whether they were indirect support requests (i.e., reduce amount of clinical duty hours/have opportunities for promotion) or direct support requests (i.e., have more spacious room or equipment/increase research budget). We then calculated variable distributions and tested for differences between genders for the two types of negotiations by using the chi-square or Fisher’s exact test. We used unadjusted and multivariable stepwise logistic regression models to estimate the odds ratios (OR) along with 95% confidence intervals (CI) for the two types of negotiation experiences. In multivariable logistic regression analyses, we analyzed the associations with adjustment for gender, age, marital status, grant amount, research type, self-rated sufficiency of research environment, position, publication, weekly working hours in patient care or research, personal burnout (PBO), work-related burnout (WBO), client (patient for the purposes of this study) related burnout (CBO), and the total of the three burnout subscales (TBO). PBO, WBO, CBO, and TBO were separately included as binary variables at the 75th percentile. Weekly working hours attributed to patient care and research were included as binary variables at the 75th percentile due to a positively skewed distribution. Age, amount of Grant-in-Aid for Young Scientists, and self-rated sufficiency of research environment were included as continuous variables.

Statistical analyses were performed using SPSS ver. 19.0 (IBM, Armonk, New York, NY, USA) and SAS ver. 9.4 (SAS institute Inc., Cary, NC, USA), and *p* < 0.05 was considered statistically significant.

## 3. Results

Table 1 shows the characteristics of KAKENHI-awarded young physician researchers, which included 101 women and 307 men. The average age of the women was 37 years, similar to that of men (*p* = not significant, NS), but women were more likely to be single (*p* < 0.001) and work in lower ranking positions (*p* = 0.001) compared to men. More than a half of the men were engaged in basic medical research while more than a half of the women were engaged in clinical medicine research (*p* = 0.028). Weekly working hours in patient care (*p* = 0.050), research (*p* = 0.013), and education (*p* < 0.001) were all longer for men than for women. The percentage of reported negotiations for “a reduction of clinical duty hours or an opportunity for promotion” was 24.0% for women and 17.4% for men, and that for “more spacious room/equipment or an increase in research budget” was 20.0% for women and 19.7% for men. Personal burnout was higher in women than in men (*p* = 0.029), but the results for the other two burnout subscales and total scores were comparable between women and men (*p* = NS).

Table 2 shows the characteristics of KAKENHI-awarded young physician researchers who did or did not have the two types of negotiation experiences. The researchers who had ever negotiated for “a reduction of clinical duty hours or an opportunity for promotion” were more likely to have higher burnout scores for the personal (*p* = 0.015), client-related (*p* = 0.003), and work-related (*p* = 0.011) dimensions and a higher total CBI (*p* = 0.004) than those who had never negotiated for these changes. The researchers who had ever negotiated for “more spacious room/equipment or an increase in research budget” were less likely to be satisfied with their clerk assistant (*p* = 0.045) and budget (*p* = 0.015) and were more likely to have higher burnout scores for the personal (*p* = 0.016), client-related (*p* = 0.014), and work-related (*p* = 0.011) dimensions and for total CBI (*p* = 0.004) than those who had never negotiated regarding these elements.

Table 3 shows the odds ratios of each covariate for the two types of negotiation experiences. The researchers who negotiated for “a reduction of clinical duty hours or an opportunity for promotion” were significantly more likely to have higher scores for PBO (OR, 2.066; 95% CI: 1.232, 3.463), CBO (OR, 2.500; 95% CI: 1.469, 4.178), WBO (OR, 1.741; 95% CI: 1.036, 2.925), and TBO (OR, 2.110; 95% CI: 1.243, 3.583) as well as 18 h of research work per week or less (OR, 2.011; 95% CI: 1.036, 3.903). The researchers who negotiated for “more spacious room/equipment or an increase in research budget” were significantly less likely to be satisfied with their clerk assistant (OR, 0.542; 95% CI: 0.326, 0.901) and research budget (OR, 0.531; 95% CI: 0.391, 0.866), while they were significantly more likely to have higher scores for CBO (OR, 1.758; 95% CI: 1.053, 2.936) and TBO (OR, 2.108, 95% CI: 1.250, 3.557).

Table 4 shows multivariable stepwise logistic regression results for the two types of negotiation experiences according to four models, adjusting for each burnout score; all models included each burnout scale one by one. The researchers who negotiated for “a reduction of clinical duty hours or an opportunity for promotion” were associated with 18 h of research work per week or less, higher burnout scores for CBO or TBO, and an increase in the unit of research budget in the model that included CBO or TBO. The researchers who negotiated for “more spacious room/equipment or an increase in research budget” were associated with older age in the model that included PBO, WBO, or TBO; single marital status in all models with each burnout scale; and higher burnout score for PBO or CBO.

## 4. Discussion

The percentage of reported negotiation for “a reduction of clinical duty hours or an opportunity for promotion” was 24.0% for women and 17.4% for men, and that “more spacious rooms/better equipment or an increase in research budget” was 20.0% for women and 19.7% for men. We demonstrated that physician researchers’ negotiations with superiors to improve their research environment were related to their mental well-being. We found that a (1) negotiation to reduce clinical hours or to have opportunities for promotions was significantly associated with a shortage of protected time for research activities, higher levels of CBO and TBO, and a unit increase of research budget; and (2) negotiation to have more spacious rooms or better equipment or to increase research budget was significantly associated with older age, single marital status, and higher levels of PBO and CBO. We discussed these findings in reference to previous studies.

Negotiation experiences for a reduction of clinical duty hours or an opportunity for promotion were associated with 18 h of research work per week or less. A recent survey with 100,000 physicians conducted by the Japanese government demonstrated that the average number of work hours for those in their 20 s was 75 h per week [22] and that the work hours become longer in specialty departments such as surgery [22] at tertiary hospitals with a severe physician shortage. Since Japanese medical doctors do not have time secured for research, it is not uncommon that some doctors readily drop out, even from scientific grant funded research. Indeed, our participants spared on average only 7–10 h a week for research, which is a very small amount of time compared to their overseas research competitors. For example, in the USA, historically, many institutions have allowed medical doctors to allocate 80% of their time for research work and 20% for clinical, teaching, and administrative work [23] together with the protection of research time by an external funding, which is officially known as the K award under the National Institute of Health grant. Alternatively, the MD-PhD combined degree program, which requires an additional 8–9 years after completing a 4-year undergraduate degree, provides the most streamlined path to becoming a physician scientist in the USA. One of the incentives for taking this path is that the National Institutes of Health (NIH) training grants, which support many of these programs, provide tuition for medical school and thereby substantially reduce student debt [24]. Although the MD-PhD program has recently been introduced in Japan’s medical education program, there are very few students who have applied to this program [25]. More efforts to arouse interest in science in young medical students are definitely required [26]. In the same model, an increase in research budget was also associated with the negotiation for a reduction of clinical duty hours and an opportunity for promotion, which suggests that the higher the grant amount the physician scientist receives, the more pressure they may perceive. This is because they have to live up to the expectation of the funding source to publish academic output in prestigious journals within a limited period of research time. This situation may also explain the client-related burnout observed in our study, indicating that these physician scientists might be frustrated by patient care, which is usually time-consuming and dominates clinical work.

Negotiating for more spacious rooms or better equipment or for an increase in research budget were related to researchers in their late 30s, of single marital status, and with higher personal or client-related burnout. The age range and single status may suggest that young and inexperienced physician scientists are more likely to have limited space, laboratory equipment, and research budget. Personal burnout in addition to client-related burnout associated with the negotiation was observed in our study. As personal burnout is characterized by emotional exhaustion and previous studies confirmed that young women in a professional position were more likely to be associated with personal burnout compared to other types of burnout [3,27,28], our findings may be explained by the characteristics of physician scientists such as young age, a lower skill level, and lower professional maturity. In addition, it should be considered that sufficient workplace support as well as space and laboratory equipment has not been provided for physician scientists as many studies have shown an association between low workplace support and emotional exhaustion [29,30,31].

Based on these results, the implications of our study include highlighting the need for regular reviews of working and research environment with physician scientists and seriously considering their requests during negotiations to ensure their mental well-being and reduce their high burnout levels. Notably, most Japanese are unwilling to negotiate with others as they are not trained in proper negotiation skills [16,32]. In addition, the perceived hierarchy of academic medical institutions can also create a difficult environment for negotiation [17]. Moreover, female academic medical faculty tend to regard negotiation as less important in their academic career than do their male counterparts [17]. Nevertheless, learning negotiation skills may be worthwhile for physician scientists under circumstances in which physician shortage is severe and no financial support for protected research time is officially available. This worst-case scenario may hold suggestions for international researchers.

To the best of our knowledge, there has been little or no previous literature on researchers’ negotiating behavior for improving their research environment and their psychological well-being. The strength of the present study is that we focused on negotiation regarding the research environment among young physician researchers and investigated whether it related to mental well-being. This study has several limitations. First, as this study was cross-sectional, the relationships found could not be interpreted as causal. In particular, the association between negotiation and burnout may be explained in two ways: (1) one might engage in negotiation because they are frustrated about their research environment; and (2) one might experience burnout because they are overwhelmed by psychological stress as a consequence of negotiation. Second, the study participation rate was relatively low. Our participants might have been dissatisfied with their research environment, while non-responders might not have been. Third, as participants were drawn from awardees of Grants-in-Aid for Young Scientists, the results may not be generalizable to physician researchers who are non-awardees. The non-awarded physicians might not exhibit the same degrees of burnout as we observed in this study because such physicians are less likely to feel pressure to publish their research output. Fourth, although our dataset did not include non-respondents, the ministry reports gender information of the total awardees of KAKENHI for Young Scientists. In 2014 and 2015, when the present study was conducted, most of the awardees were men (i.e., 75% and 74.5%), with the rest women. The gender ratio of total awardees was close to the ratio of our participants (i.e., 101 women/307 men), suggesting that our sample was representative of general awardees of KAKENHI for Young Scientists. A further prospective study is warranted to clarify the associations between negotiation and researchers’ performance and psychological well-being.

## 5. Conclusions

In the present study, we examined the association between negotiations for improving research environments and mental well-being among young physician researchers in Japan. About one-fifth of young physician researchers reported experience in negotiating. Negotiating for a reduction of clinical duty hours or an opportunity for promotion was associated with 18 h of research work per week or less. Negotiating for more spacious rooms or better equipment or for an increase in research budget was associated with researchers in their late 30s, of single marital status, and with higher personal or client-related burnout. It is important for young physician researchers to negotiate with their superiors to gain ideal research environments, to improve psychological well-being, which is fundamentally important for research performance.

## Figures and Tables

**Table 1:** Characteristics of Grant-in-Aid for Scientific Research (KAKENHI) awarded young physician researchers in Japan.

	Female (*n* = 101)	Male (*n* = 307)	p *
Age, mean ± SD	36.6 ± 2.5	36.8 ± 2.7	0.585
Marital status, *n* (%)			<0.001
Married	71 (70.3)	281 (91.5)	
Single (single/divorced)	30 (29.7)	26 (8.5)	
Amount of Grant-in-Aid for Young Scientists, median (25%, 75%)	364.000 (290.000, 400.000)	377.000 (300.000, 403.000)	0.149
Research type, *n* (%)			0.028
Basic medicine	41 (42.3)	170 (55.6)	
Clinical medicine	51 (52.6)	114 (37.3)	
Others	5 (5.2)	22 (7.2)	
Position, *n* (%)			0.001
Associate professor	2 (2.0)	3 (1.0)	
Lecturer	4 (4.0)	36 (11.7)	
Assistant professor	59 (58.4)	164 (53.4)	
Administrator/director	1 (1.0)	29 (9.5)	
Registered doctor	27 (26.3)	62 (20.2)	
Resident/Research associate/Others	8 (7.9)	13 (4.2)	
Weekly working time, median (25%, 75%)			
Patient care	28.000 (19.250, 40.000)	35.000 (22.500, 46.800)	0.050
Research	7.420 (4.000, 12.375)	10.000 (5.000, 18.750)	0.013
Education and Career/Teaching/Administration	8.000 (4.000, 13.500)	12.250 (7.000, 21.000)	<0.001
Self-rated sufficiency of research environment **, mean ± SD			
Space	3.2 ± 1.0	3.1 ± 1.1	0.561
Equipment	3.4 ± 1.0	3.2 ± 1.1	0.040
Clerk assistant	2.7 ± 1.1	2.6 ± 1.1	0.232
Budget	3.3 ± 0.9	3.1 ± 1.0	0.152
Negotiation, *n* (%)			
Decrease of clinical duty hours/Promotion opportunity	24 (24.0)	53 (17.4)	0.143
Have more spacious rooms or equipment/Increase research budget	20 (20.0)	60 (19.7)	0.943
Copenhagen burnout inventory (CBI), mean ± SD			
Personal burnout (points), alpha *** = 0.856	41.9 ± 20.5	36.7 ± 20.8	0.029
Client-related burnout (points), alpha *** = 0.816	28.8 ± 20.5	27.8 ± 17.3	0.630
Work-related burnout (points), alpha *** = 0.844	30.1 ± 18.4	28.9 ± 17.8	0.536
Total (points)	100.8 ± 53.3	93.4 ± 49.1	0.194
Research paper, median (25%, 75%)			
Original paper	2.00 (0.00, 2.50)	2.00 (1.00, 4.00)	0.126
Review	0.00 (0.00, 0.00)	0.00 (0.00, 0,00)	0.259
All	1.00 (0.00, 2.00)	2.00 (1.00, 4.00)	0.068

* Based on chi-square test or Fisher’s exact test. ** From 1 for strongly disagree to 4 strongly agree. *** Alpha indicates Cronbach’s alpha.

**Table 2:** Characteristics of KAKENHI-awarded young physician researchers who did or did not have the two types of negotiation experiences.

	Reduce Amount of Clinical Duty Hours/Have Promotion Opportunity		p *	Have More Spacious Rooms or Better Equipment/Increase Research Budget		p *
	Negotiation (+)	Negotiation (-)		Negotiation (+)	Negotiation (-)	
Sex, *n* (%)			0.143			0.943
Male	53 (68.8%)	252 (76.8%)		60 (75.0%)	245 (75.4%)	
Female	24 (31.2%)	76 (23.2%)		20 (25.0%)	80 (24.6%)	
Age, mean ± SD	36.97 ± 2.33	36.72 ± 2.67	0.436	37.19 ± 2.59	36.66 ± 2.60	0.106
Marital status, *n* (%)			0.590			0.061
Married	68 (88.3%)	282 (86.0%)		64 (80.0%)	286 (88.0%)	
Single (single/divorced)	9 (11.7%)	46 (14.0%)		16 (20.0%)	39 (12.0%)	
Amount of Grant-in-Aid for Young Scientists, median (25%, 75%)	370.5	377	0.279	377	377	0.161
	(280.0, 390.0)	(310.0, 403.0)		(300.0, 403.0)	(300.0, 400.0)	
Research type, *n* (%)			0.763			0.203
Basic medicine	39 (51.3%)	172 (53.1%)		46 (58.2%)	165 (51.4%)	
Clinical medicine	33 (43.4%)	129 (39.8%)		31 (39.2%)	131 (40.8%)	
Others	4 (5.3%)	23 (7.1%)		2 (2.5%)	25 (7.8%)	
Self-rated sufficiency of research environment, mean ± SD						
Space	3.14 ± 1.14	3.14 ± 1.10	0.968	2.95 ± 1.05	3.18 ± 1.12	0.089
Equipment	3.16 ± 1.11	3.23 ± 1.08	0.580	3.01 ± 1.05	3.27 ± 1.09	0.059
Clerk assistant	2.43 ± 1.13	2.62 ± 1.09	0.170	2.36 ± 1.08	2.64 ± 1.09	0.045
Budget	3.10 ± 1.07	3.17 ± 1.00	0.603	2.91 ± 1.05	3.22 ± 1.00	0.015
Weekly working hours, median (25%, 75%)						
Patient care	32.612	32	0.783	29	32.5	0.207
	(23.063, 45.094)	(20.0, 45.0)		(15.785, 47.250)	(22.5, 45.0)	
Research	6	10	0.279	10	9	0.264
	(3.813, 12.000)	(5.0, 18.0)		(5.00, 19.40)	(4.50, 16.80)	
Others	13.35	11	0.285	11.11	11	0.303
	(5.105, 25.000)	(6.0, 19.5)		(6.813, 24.750)	(6.00, 20.00)	
Position, *n* (%)			0.288			0.483
Associate professor/lecturer/administrator/director	11 (14.3%)	64 (19.5%)		17 (21.3%)	58 (17.8%)	
Others	66 (85.7%)	264 (80.5%)		63 (78.8%)	267 (82.2%)	
Publications			0.937			0.117
2≤	36 (46.8%)	155 (47.3%)		44 (55.0%)	147 (45.2%)	
1≥	41 (53.2%)	173 (52.7%)		36 (45.0%)	178 (54.8%)	
CBI, mean ± SD						
PBO	43.18 ± 21.72	36.82 ± 20.37	0.015	43.02 ± 22.16	36.81 ± 20.24	0.016
CBO	34.93 ± 22.72	26.52 ± 16.54	0.003	33.19 ± 20.82	26.88 ± 17.24	0.014
WBO	33.86 ± 19.62	28.13 ± 17.34	0.011	33.75 ± 20.13	28.10 ± 17.17	0.011
Total	111.97 ± 57.09	91.48 ± 47.73	0.004	109.96 ± 56.14	91.79 ± 48.06	0.004

* Based on the chi-square test or Fisher’s exact test.

**Table 3:** Odds ratios of each covariate for the two types of negotiation experiences.

			Reduce Amount of Clinical Duty Hours/Have Promotion Opportunity		Have More Spacious Rooms or Equipment/Increase Research Budget	
			OR	95%CI	OR	95%CI
Sex						
	Female		1.501	0.870–2.593	1.021	0.580–1.797
	Male		1.000		1.000	
Age, years		37≤	1.396	0.833–2.341	1.152	0.698–1.901
Marital status						
	Single		0.811	0.379–1.738	1.833	0.965–3.484
Amount of Grant-in-Aid for Young Scientists		377≤	0.825	0.498–1.367	1.066	0.644–1.766
Research type						
	Basic medicine		1.304	0.427–3.985	3.485	0.796–15.261
	Clinical medicine		1.471	0.476–4.547	2.958	0.665–13.158
	Others		1.000		1.000	
Self-rated sufficiency of research environment						
	Space	3≤	1.123	0.662–1.904	0.699	0.423–1.154
	Equipment	3≤	0.943	0.548–1.624	0.752	0.445–1.269
	Clerk assistant	3≤	0.716	0.433–1.186	0.542	0.326–0.901
	Budget	3≤	0.753	0.443–1.280	0.531	0.319–0.886
Position						
	Associate professor/lecturer/administrator/director		0.688	0.343–1.377	1.242	0.677–2.278
	others		1.000			
Publications						
		2≤	0.980	0.596–1.612	1.480	0.905–2.420
CBI *						
	PBO	50.00≤	2.066	1.232–3.463	1.667	0.996–2.792
	CBO	37.50≤	2.500	1.496–4.178	1.758	1.053–2.936
	WBO	39.29≤	1.741	1.036–2.925	1.508	0.899–2.528
	Total	126.04≤	2.110	1.243–3.583	2.108	1.250–3.557
Weekly working hours in research **		<18	2.011	1.036–3.903	0.984	0.559–1.733
Weekly working hours in patient care **		45≤	0.819	0.458–1.465	0.912	0.520–1.600

* Divided into binary variable at the 75th percentile. ** Divided into binary variable at the 75th percentile due to positive skewed distribution.

**Table 4:** Multivariablef stepwise logistic regression results for the two types of negotiation experiences according to 4 models adjusting for each burnout score; all models included each burnout scale, one at a time.

	4 Models Adjusting for Each Burnout Score *			
	Model for PBO	Model for CBO	Model for WBO	Model for TBO
	OR (95%CI)	OR (95%CI)	OR (95%CI)	OR (95%CI)
18 h of research work or less per week	2.208 (1.111–4.389)	2.498 (1.196–5.215)	2.208 (1.111–4.389)	2.568 (1.236–5.334)
Higher burnout score (score above the 75th percentile) of each CBI subscale	-	2.615 (1.478–4.625)	-	2.076 (1.187–3.630)
Research budget (a unit of 10,000 JPY)	-	1.001 (1.000–1.002)	-	1.001 (1.000–1.002)
Age	1.117 (1.006–1.240)	-	1.118 (1.007–1.242)	1.118 (1.007–1.242)
Single marital status	2.568 (1.301–5.071)	2.273 (1.165–4.432)	2.509 (1.278–4.926)	2.509 (1.278–4.926)
Higher burnout score (score above the 75th percentile) of each CBI subscale	1.774 (1.014–3.103)	3.064 (1.506–6.232)	-	-

* gender, age, marital status, amount of grant, research type, self-rated sufficiency of research environment (space, equipment, administrative support, and budget), position, publication, personal burnout (PBO), client related burnout (CBO), work-related burnout (WBO), weekly working hours in research, and in patient care were included in multivariable stepwise logistic analysis model for each negotiation experience separately.
